# Vacancy impacts on electronic and mechanical properties of MX2 (M = Mo, W and X = S, Se) monolayers†

**DOI:** 10.1039/d3ra00205e

**Published:** 2023-02-24

**Authors:** Seyedeh Alieh Kazemi, Sadegh Imani Yengejeh, Samuel Akinlolu Ogunkunle, Lei Zhang, William Wen, Alan Wee-Chung Liew, Yun Wang

**Affiliations:** a Centre for Catalysis and Clean Energy, School of Environment and Science, Griffith University Gold Coast Campus QLD 4222 Australia yun.wang@griffith.edu.au; b School of Information and Communication Technology, Griffith University Gold Coast Queensland 4215 Australia

## Abstract

Monolayers of transition metal dichalcogenides (TMD) exhibit excellent mechanical and electrical characteristics. Previous studies have shown that vacancies are frequently created during the synthesis, which can alter the physicochemical characteristics of TMDs. Even though the properties of pristine TMD structures are well studied, the effects of vacancies on the electrical and mechanical properties have received far less attention. In this paper, we applied first-principles density functional theory (DFT) to comparatively investigate the properties of defective TMD monolayers including molybdenum disulfide (MoS_2_), molybdenum diselenide (MoSe_2_), tungsten disulfide (WS_2_), and tungsten diselenide (WSe_2_). The impacts of six types of anion or metal complex vacancies were studied. According to our findings, the electronic and mechanical properties are slightly impacted by anion vacancy defects. In contrast, vacancies in metal complexes considerably affect their electronic and mechanical properties. Additionally, the mechanical properties of TMDs are significantly influenced by both their structural phases and anions. Specifically, defective diselenides become more mechanically unstable due to the comparatively poor bonding strength between Se and metal based on the analysis of the crystal orbital Hamilton population (COHP). The outcomes of this study may provide the theoretical knowledge base to boost more applications of the TMD systems through defect engineering.

## Abstract 2

The impacts of six different types of vacancies on the electronic and mechanical properties of MX2 (M = Mo, W and X = S, Se) monolayers have been theoretically revealed.

## Graphical abstract

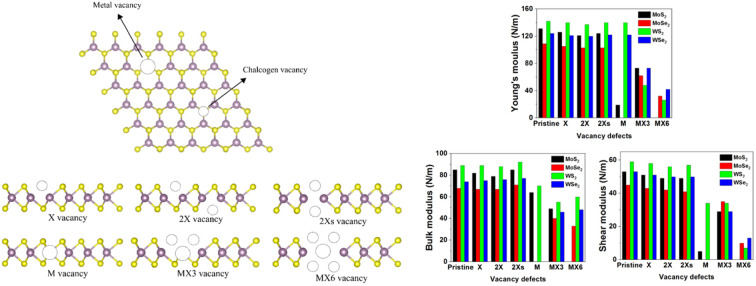

## 1. Introduction

Transition metal dichalcogenides (TMDs) have attracted tremendous attention as they exhibit outstanding physical and chemical characteristics.^1–6^ Their two-dimensional (2D) structures possess the chemical formula of MX2 (M represents a transition metal atom, *e.g.* molybdenum (Mo) or tungsten (W), and X represents a chalcogen atom, *e.g.* sulfur (S) or selenium (Se)) with a strong planar covalent bonding among the X–M–X atoms in the latitude dimension and comparatively weak van der Waals (vdW) interactions between the layers in the layered-structured configurations,^7^ therefore, TMD monolayers can be easily manufactured through mechanical or liquid exfoliation.^8,9^ These monolayers play increasingly significant roles in theoretical and practical applications due to possessing unique two-dimensional structures, extensive chemical compositions, and various material properties from semiconductors, and metals to superconductors.^1,4–6^ However, structural vacancies are inevitably formed during the synthesis of monolayer TMDs due to the imperfection of the growth process or mechanical manipulation.^10^ Lin *et al.* used scanning transmission electron microscopy (STEM) to find the M vacancies in the filtered annular dark-field (ADF) images.^11^ Zhang *et al.* applied low-temperature scanning tunneling microscopy (STM) and spectroscopy to identify the single W vacancies in WSe_2_.^12^ When the MoS_2_ monolayer grew on the SiO_2_ substrate using the chemical vapor deposition method, Zhou *et al.* found both X and M-based vacancies in the ADF-images.^13^ Since the electronic and mechanical properties of 2D materials are strongly linked to their potential applications, the characterizations of such TMD monolayers with vacancies are imperative.

**Fig. 1:**
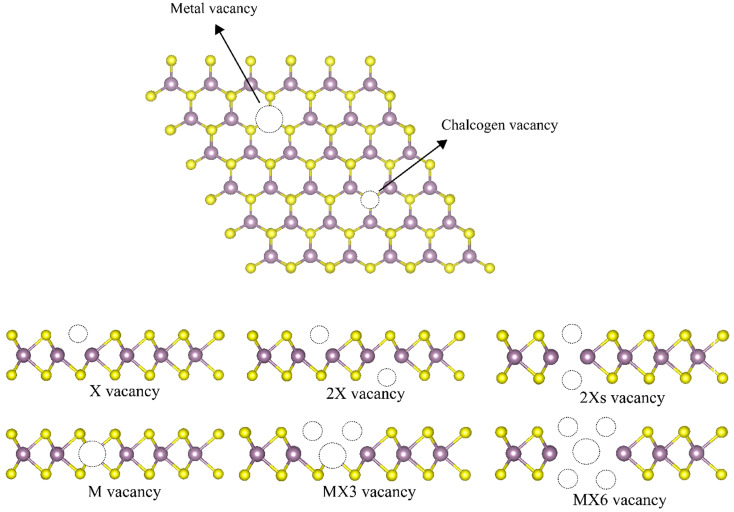
Illustration of different point vacancy defects in TMD monolayers.

Among different experimental measurements,^13–15^ there are six types of point defect, *i.e.* monochalcogenide vacancy (X vacancy), dichalcogenide vacancy (2X vacancy), aligned dichalcogenide vacancy (2Xs vacancy) metal vacancy (M vacancy), vacancy complex of M and nearby three chalcogens (MX3 vacancy), and vacancy complex of M and nearby three chalcogen pairs (MX6 vacancy) are considered in this study as illustrated in Fig. 1. The monochalcogenide vacancy, defined as a single missing chalcogenide anion, is the most common point defect structure, widely observed in experiments as it has the lowest formation energy.^13^ It was reported that monosulfide vacancies exist primarily in the bottom layer of the MoS_2_ membrane under irradiation.^16^ Furthermore, the 2Xs vacancy, which is defined as a missing pair of S atoms aligned the *c*-axis of the MoS_2_ lattice, can also form.^13^ The 2X vacancy systems were also probed to simulate the single missing atom from each top and bottom side, whose placements are not aligned with the *c*-axis of the TMD monolayer lattice.^13^ A recent experimental study reveals that the metal vacancy may form in 2D nanosheets, which can greatly change the reactivities of neighboring atoms.^12,17^ In addition, previous investigations have suggested that the metal complex vacancies, *e.g.*, MX3 and MX6, may be formed after the TMD monolayer was exposed to electron irradiation.^13^

Recently, the impact of the vacancies on the mechanical and electronic properties of some TMD monolayers have been theoretically investigated. For example, Cao *et al.* found that the anion vacancy combined with the strain engineering can be used to manipulate the mechanical, electronic and optical properties of 1H WS_2_.^18^ Bahmani *et al.* systematically studied both anion vacancies and metal complex vacancies on the mechanical and electronic properties of 1H MoS_2_. They found that the strain can significantly modify the defect level inside the bandgap and their orbital characteristics.^19^ Both studies support that the synergic effects of the point vacancies and the mechanical deformation. However, up to our knowledge, there is no comparative study on the electronic and mechanical properties of TMDs affected by different point defect effects.

In this study, the defect impacts on the electronic and mechanical properties of most studied 1H TMDs including MoS_2_, MoSe_2_, WS_2_, and WSe_2_ monolayers were investigated by the means of density functional theory (DFT) calculations. The crystal orbital Hamilton population (COHP) analysis was conducted to investigate the bonding strength between the M–X atoms near the vacancies to further understand the impact of vacancies on the properties of the TMDs. Our results suggested that only the metal and metal complex vacancy defects significantly affect the electronic and mechanical properties of the TMD monolayers.

## 2. Computational details

In this study, all DFT calculations were carried out using the projector augmented wave (PAW) method using the Vienna *ab initio* simulation package (VASP) algorithm.^20–22^ The generalized gradient approximation (GGA) with the format of Perdew–Burke–Ernzehof (PBE) was used for the exchange-correlation functional.^23^ Electron-ion interactions were described using PAW potentials,^24^ with valence configurations of 4s^2^4p^6^5s^1^4d^5^ for Mo (Mo_sv), 5s^2^5p^6^6s^1^5d^5^ for W (W_sv), 3s^2^3p^4^ for S (S), and 4s^2^4p^4^ for Se (Se). The DFT-D3 approach was applied in this research in consideration of the influence of the van der Waals force.^25–30^ The DFT-D3 method was chosen based on our previous studies on the same TMD monolayers, which demonstrates that the DFT-D3 results can reproduce the experimental data and outperform the values from LDA, PBE and DFT-D2 methods.^7^ A plane-wave basis set with a cut-off kinetic energy of 520 eV was employed to expand the smooth part of the wave function.

A (3 × 3) pristine TMD monolayer cell was used to build the atomistic models of the defective systems. The vacancies were generated by removing the relevant atoms to keep the system charge neutral, as shown in Fig. 1. Prior to the calculation of the mechanical characteristics of the TMDs, both the lattice constants and the atomic coordinates were optimized. All the atoms were allowed to relax until the Hellmann–Feynman forces are smaller than 0.02 eV Å^−1^, and the convergence criterion for the self-consistent electronic optimization loop was set to 1 × 10^−5^ eV. The gamma-cantered *k*-point meshes with a reciprocal space resolution of 0.04 × 2π/Å was utilized for the structural optimization. A denser gamma-cantered *k*-point meshes with a reciprocal space resolution of 0.02 × 2π/Å was employed for the analysis of electronic properties. To investigate the elastic constants of the TMDs according to the generalized Hooke's law, the energies as a function of strain (*ε*) in the strain range −2.0% ≤ *ε* ≤ 2.0% with an increment of 0.5% were calculated. The elastic constants *C*_*ij*_ were obtained by fitting a second-order polynomial to the change on the total energy *versus* applied strain. The Young's and shear moduli and Poisson's ratio were calculated using the same method explained in our previous studies in detail.^2,31,32^ The data were obtained from post-processing the DFT results using the VASPKIT code.^33^

## 3. Results and discussion

### 3.1 Structural properties

The geometrical structures of TMDs and their structural defects are illustrated in Fig. 1. The calculated lattice constants, monolayer thickness, M–X bond length, and X–M–X bond angles of all 2D systems are listed in Table 1. The monolayer thickness is defined as the maximum height difference between X anions. Our findings from the pure 1H TMDs are consistent with the values that have already been reported.^34–37^ The introduction of point vacancy defects changes the lattice constants, layer thickness and bond length between atoms next to the vacancies. Most of the lattice constants shrink after the formation of the vacancies. The formation of M vacancies has quite little influence on the lattice constants, which are even slightly longer than that of the pristine TMD monolayers. Additionally, the formation of the vacancies leads to the corrugated structure as evidenced by the increased thickness of monolayers. Disulfide monolayers with M vacancies are unique since they almost exactly match those of pristine monolayers, whereas diselenides with M vacancies result in more corrugated monolayers. Interestingly, the thickness of the monolayer with the 2Xs are generally smaller than that of X and 2X, which can be ascribed to the higher symmetry of the monolayers with the 2Xs vacancies. The bond length reduces after introducing the defects, where the MX3 vacancy defect exhibits the largest difference in the value of the bond length.

**Table 1:** Calculated lattice constants *a* (Å) and *b* (Å), monolayer thickness *t* (Å), M–X bond length *d* (Å) by using a (3 × 3) TMD monolayer cell

1H-TMD	Defect	*a* (Å)	*b* (Å)	*t* (Å)	*d* (Å)
MoS_2_	Pristine	9.51	9.51	3.13	2.41
	X	9.41	9.41	3.23	2.37
	2X	9.32	9.32	3.30	2.37
	2Xs	9.31	9.31	3.16	2.39
	M	9.52	9.52	3.13	2.37
	MX3	9.46	9.46	3.23	2.27
	MX6	9.04	9.04	3.27	2.36
MoSe_2_	Pristine	9.87	9.87	3.35	2.54
	X	9.78	9.78	3.51	2.50
	2X	9.63	9.63	3.63	2.50
	2Xs	9.66	9.66	3.40	2.52
	M	9.88	9.88	3.45	2.52
	MX3	9.81	9.81	3.47	2.41
	MX6	9.24	9.24	3.64	2.49
WS_2_	Pristine	9.54	9.54	3.15	2.42
	X	9.43	9.43	3.29	2.39
	2X	9.32	9.32	3.40	2.38
	2Xs	9.33	9.33	3.17	2.40
	M	9.57	9.57	3.15	2.37
	MX3	9.46	9.46	3.25	2.28
	MX6	9.09	9.09	3.33	2.37
WSe_2_	Pristine	9.87	9.87	3.38	2.54
	X	9.76	9.76	3.59	2.52
	2X	9.63	9.63	3.77	2.51
	2Xs	9.66	9.66	3.41	2.52
	M	9.90	9.90	3.52	2.53
	MX3	9.79	9.79	3.50	2.42
	MX6	9.35	9.35	3.69	2.49

### 3.2 Mechanical properties

**Fig. 2:**
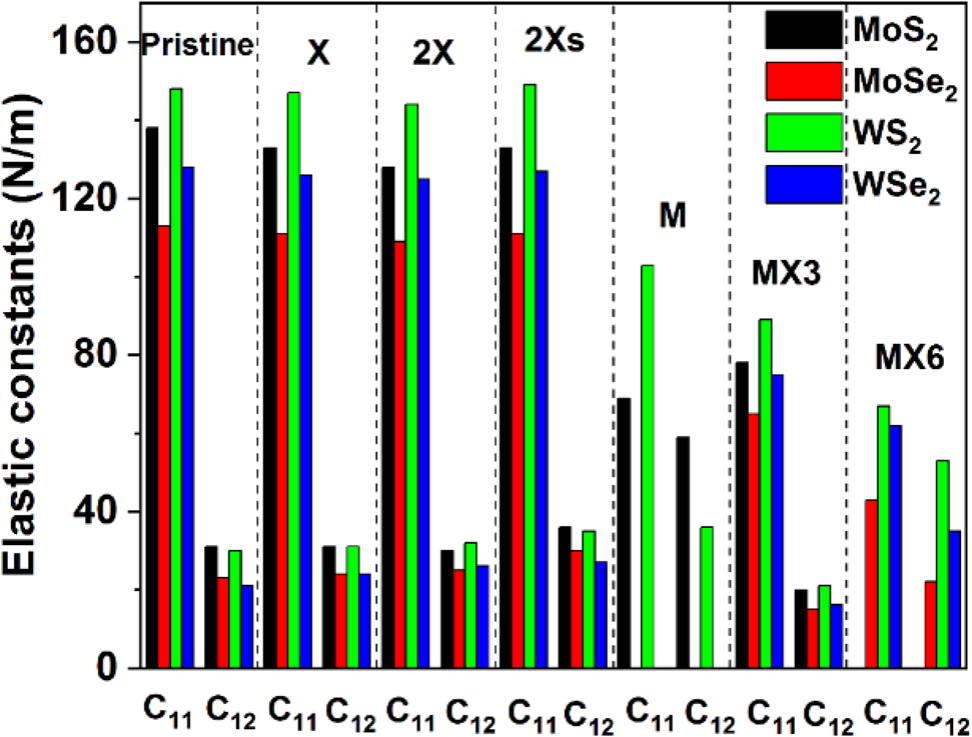
Elastic constants of the defective TMDs.

**Fig. 3:**
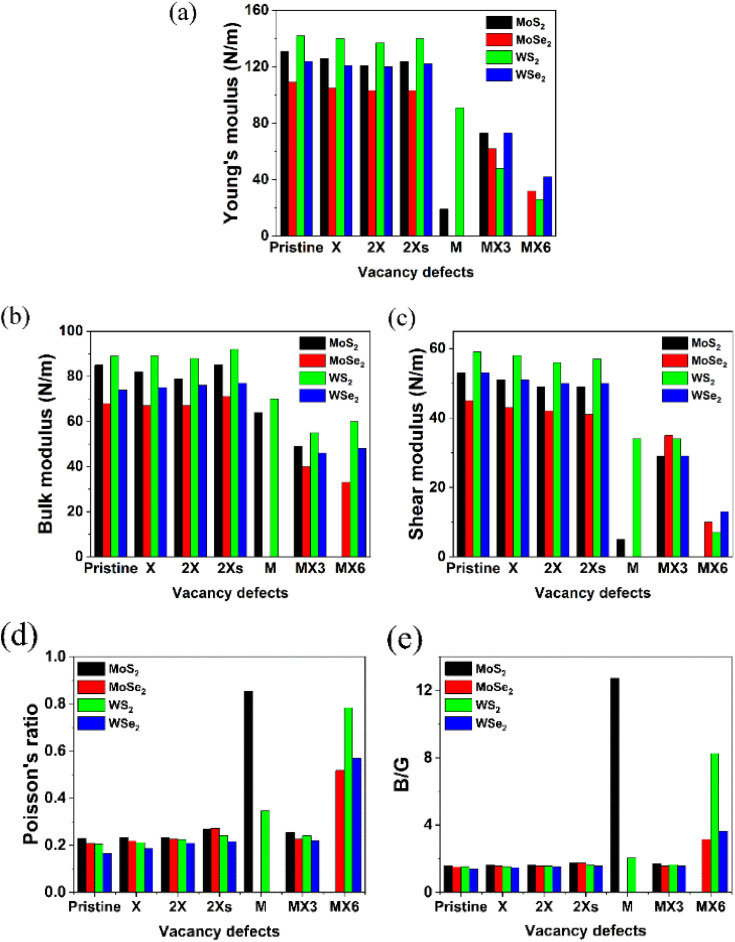
Different mechanical properties of the defective TMDs.

The calculated elastic constants and mechanical properties (including Young's and shear moduli and Poisson's ratio) of different 1H TMD monolayers are shown in Fig. 2 and 3. The elastic constants of the W-based TMD monolayers are higher than those of the Mo-based monolayers. The smallest elastic constant values are found in MoSe_2_. Disulfides have greater elastic constants in comparison with diselenides among the investigated material models without or with various forms of vacancies. It suggests that the X anions has a stronger impact on the mechanical properties of TMDs, which matches the conclusion from previous studies.^38^

Fig. 2 indicates that the impact of the X, 2X and 2Xs vacancies on the elastic constants of the structure is small, as evidenced by the negligible change of the *C*_11_ and *C*_12_ values. The introduction of the X vacancy can reduce the *C*_11_ and *C*_12_ slightly, which are further decreased when the coverage of X vacancy increases to 2X and 2Xs vacancies. Notably, the influence of the 2X vacancy on *C*_11_ and *C*_12_ is stronger than the effect of the 2Xs vacancy, which shows that the coverage, position, and symmetry of X vacancies can change the elastic constants while having limited effects on them. Comparatively, the formation of metal and metal complex vacancies can greatly alter the elastic constants. MoSe_2_ and WSe_2_ show mechanical instability when M vacancy is introduced to the configuration of the model. Furthermore, MoS_2_ becomes mechanically unstable when the MX6 vacancy is formed, as a result, the *C*_*ij*_ values of these systems are not provided in Fig. 2. Moreover, the formation of M and MX3 and MX6 vacancies leads to a decrease in the values of the elastic constants, particularly the *C*_11_. Based on the available data, the impact of MX6 on the *C*_11_ is the largest among three metal-related vacancies. M vacancy has the smallest the impact in comparison with MX3 and MX6 vacancies. However, the elastic constants still follow the same trend of that of the pristine TMD monolayers. The disulfides have large *C*_*ij*_ values than the diselenides. And the W-based TMDs are stronger than the Mo-based TMDs.

Based on the elastic constants, the Young's moduli (*Y*), bulk moduli (*B*), shear moduli (*G*), Poisson's ratio and *B*/*G* ratio for the pristine and defective TMD monolayers were calculated and shown in Fig. 3. Similar as the elastic constants, all the disulfides without or with vacancies possess the larger values for Young's, bulk and shear moduli. The W-based TMDs also have the larger Young's, bulk and shear moduli than Mo-based TMDs. The coverage, location and symmetry of the X vacancies can change their Young's, bulk and shear moduli. However, the influence of the X vacancies on the mechanical strength is small. As a comparison, the formation of M and metal complex vacancies significantly changes the mechanical strength, especially, the M vacancy defect makes the diselenide TMDs mechanically unstable. Further, MoS_2_ monolayers with the M vacancies exhibit the lowest *Y* and *G* values compared to MoS_2_ with other types of vacancy defects. The MX6 and MX3 vacancies have the largest impact on *Y*, *B* and *G* moduli of TMD monolayers. The 1H MoS_2_ becomes unstable after introducing the MX6 vacancy defect. Likewise, the *Y* and *G* moduli of TMD monolayers with MX6 vacancy are much smaller than those with other types of vacancies.

Contrarily, the Poisson's ratios and *B*/*G* ratios increases greatly after the introduction of M and MX6 vacancies, as shown in Fig. 3d and e. The TMDs with other types of the vacancies comparatively show the similar values for the Poisson's ratio, which is in the range of 0.19 to 0.23. For MoS_2_ and WS_2_ with the M vacancy, the Poisson's ratio is 0.854 and 0.345, while TMD monolayers with the MX6 vacancies, the Poisson's ratios are higher than 0.5. The Poisson's ratio defines the ratio of transverse strain to the axial strain. The high Poisson's ratio of TMDs with the M and MX6 vacancies suggests that these defective TMDs become mechanically softer and corresponds with the change of *B*/*G* ratios. The ratio of *B*/*G* is used to distinguish ductility and brittleness of a material. The critical value that separates ductile and brittle materials is around 1.75. The material with a *B*/*G* ratio lower than 1.75 behaves in a brittle manner. As shown in Fig. 3e, the formation of M and MX6 vacancies leads to much higher *B*/*G* ratios in term to the critical value of 1.75, which suggest that the TMDs with the M and MX6 vacancies become more ductile. While MX3 vacancies are also one of the metal complex vacancies, they show the similar Poisson's ratio and *B*/*G* ratio values to that of the pristine TMDs and TMDs with X, 2X and 2Xs vacancies. It demonstrates that the formation of the MX3 vacancy has relatively small impact of the hardness and brittleness of TMD monolayers here while it can greatly reduce the *Y*, *B* and *G* moduli of TMDs. Alternately, the formation of MX3 vacancies has the least influence on the mechanical stabilities, as evidenced by the change of the mechanical properties shown in Fig. 3. This matches previous experimental studies, which reveals that X and MX3 vacancies are dominant after the electron irradiation.^13^ Consequently, the physicochemical properties of TMDs with the X and MX3 vacancies are further understood by their impact on the other physicochemical properties of TMDs.

### 3.3 X and MX3 vacancies

Firstly, the coverage impact of the X and MX3 vacancies have been investigated. It was discovered that the growth of the X vacancies has a relatively minimal effect, as evidenced by the change in the mechanical properties of TMDs with X, 2X, and 2Xs vacancies, thus the coverage effect of the X vacancies on the mechanical properties is not high. To investigate the coverage effect of the MX3, a large cell based on the (9 × 9) pristine cell was used. Table 2 lists the elastic constant and mechanical properties of 1H-MoS_2_ with MX3 vacancy defect in its (3 × 3) and (9 × 9) unit cells. The similar mechanical properties of the models indicating that the MX3 coverage has little effect on the mechanical features of the TMDs when the unit cell is large than (3 × 3).

**Table 2:** Elastic constants and mechanical properties of MX3 defective 1H-MoS_2_ at different coverages

TMD	Vacancy	Unit cell	*C*_11_	*C*_12_	*Y*	*B*	*G*
MoS_2_	MX3	(3 × 3)	78.3	20.0	73.2	49.2	29.2
		(9 × 9)	78.7	19.9	73.7	49.3	29.4

**Fig. 4:**
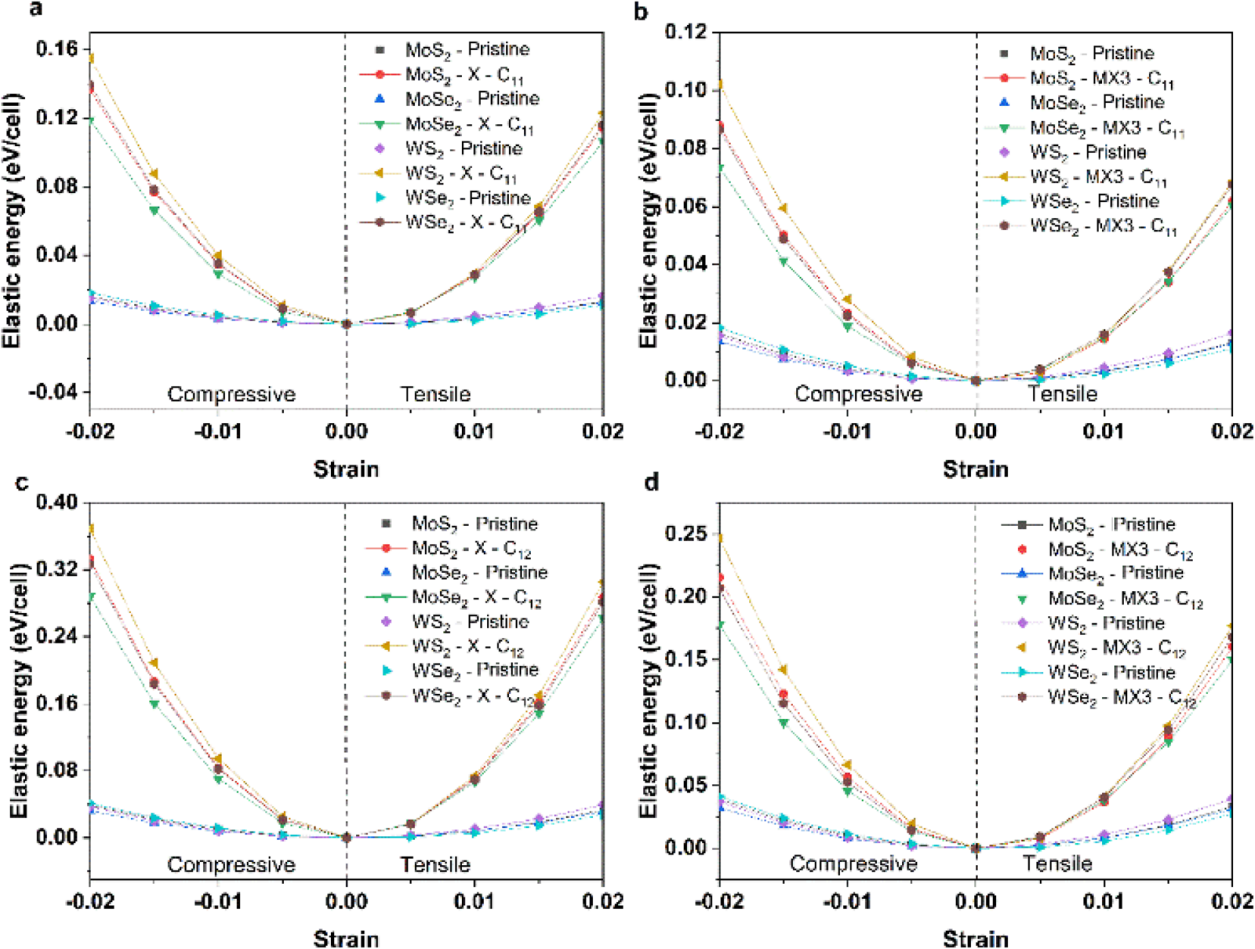
Elastic energies of (a) pristine and TMDs with X vacancies under the compression and tension along the *x* uniaxial direction; (b) pristine and TMDs with MX3 vacancies under the compression and tension along the *x* uniaxial direction; (c) pristine and TMDs with X vacancies under the compression and tension along the *xy* biaxial direction; and (d) pristine and TMDs with MX3 vacancies under the compression and tension along the *xy* biaxial direction.

To understand the impact of X and MX3 vacancies on the mechanical properties, the elastic energies of the TMDs under the compression and tension along 11 and 12 directions have been calculated and shown in Fig. 4. The elastic energies of TMD monolayers with the X and MX3 vacancies are significantly higher than those of pure TMDs. The elastic energies of the TMDs with the X vacancies are larger than those of the MX3 vacancies. The highest and smallest changes occur in the WS_2_ and MoSe_2_ materials, respectively. These results are consistent with the trend of the elastic constants and mechanical moduli. However, the elastic energies along the *x* uniaxial direction are lower than those along the *xy* biaxial direction, indicating that the creation of various vacancies may alter the strength of the bonds between the atoms nearby.

**Fig. 5:**
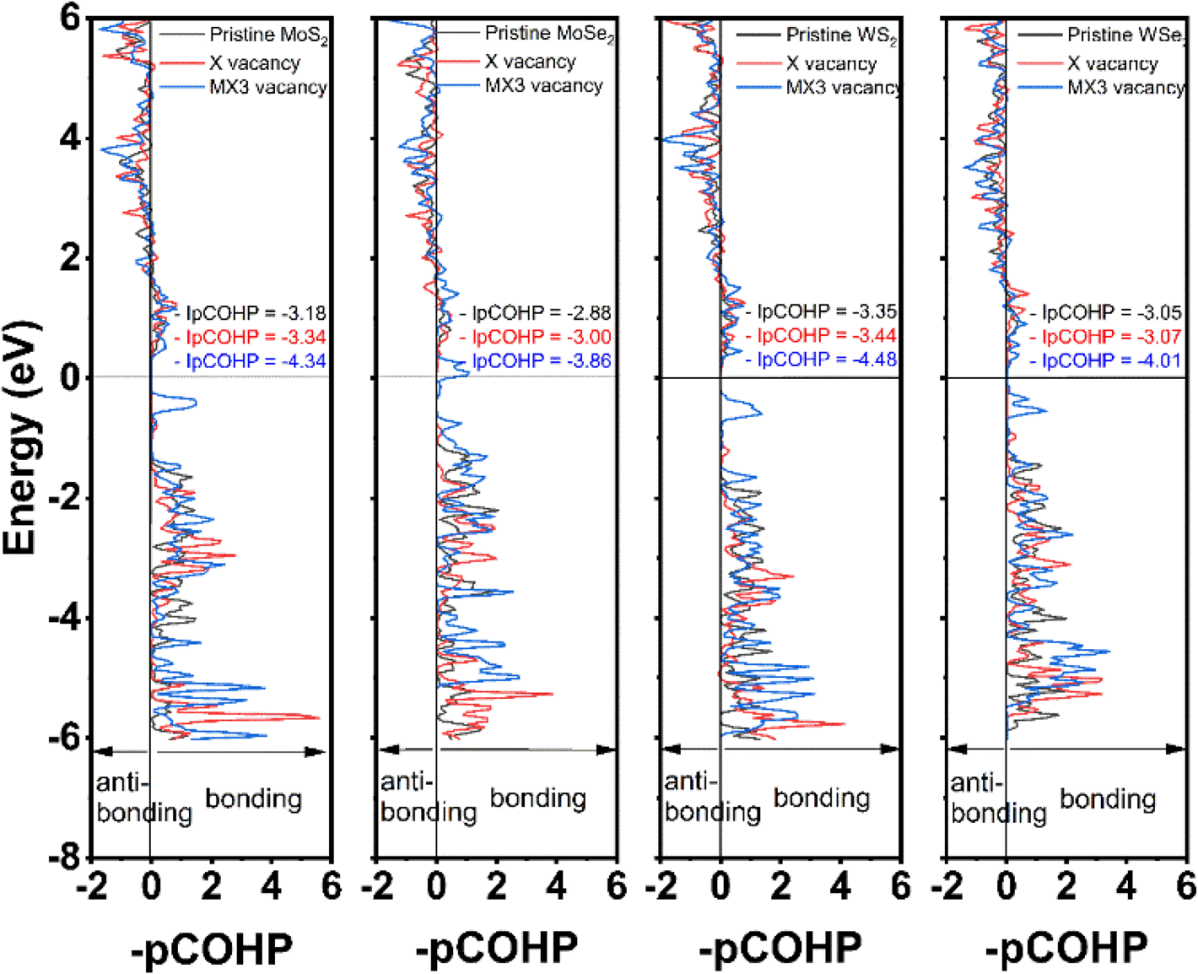
The calculated –pCOHP and –IpCOHP of the shortest M–X bond in defective monolayer TMDs.

The partial crystal orbital Hamilton population (–pCOHP) of the bond between M and X atoms next to the vacancies was analyzed in this study using LOBSTER program. The –pCOHP is through the partition of the band-structure energy into the orbital–pair interactions, which can be used to understand the bonding strength between M and X atoms. The –pCOHP indicates the bonding and antibonding interaction between atoms.^39,40^ The bonding and antibonding mechanisms can be characterized based on the negative and positive overlap population, respectively. The bond strengths between M and X atoms are quantitatively determined by taking the integral of –pCOHP (–IpCOHP) up to the Fermi level, which are also provided in Fig. 5. The lower –IpCOHP value suggests a stronger covalent bonding strength between the atoms. Our results demonstrate that –IpCOHP values of W–S bonds are the smallest among TMDs with the same type of the vacancies, while the Mo–Se bonds have the highest –IpCOHP values. The bonding strength is responsible to the trend of the elastic constants and mechanical moduli observed in Fig. 2 and 3. The –pCOHP analysis results reveal that the change of the bonding characteristics after the formation of X vacancies is small, thus detailing the small impact of X vacancies on the mechanical properties of TMD monolayers. Furthermore, the M–X bond nearest to the MX3-vacancy has the strongest bonding strength, which shows that the MX3 has a greater impact on the local mechanical strength than is indicated by the observed increased mechanical strength. As a consequent, the change of the mechanical properties of the TMD monolayers with the MX3 vacancy is also larger in comparison to the one with X vacancies.

**Fig. 6:**
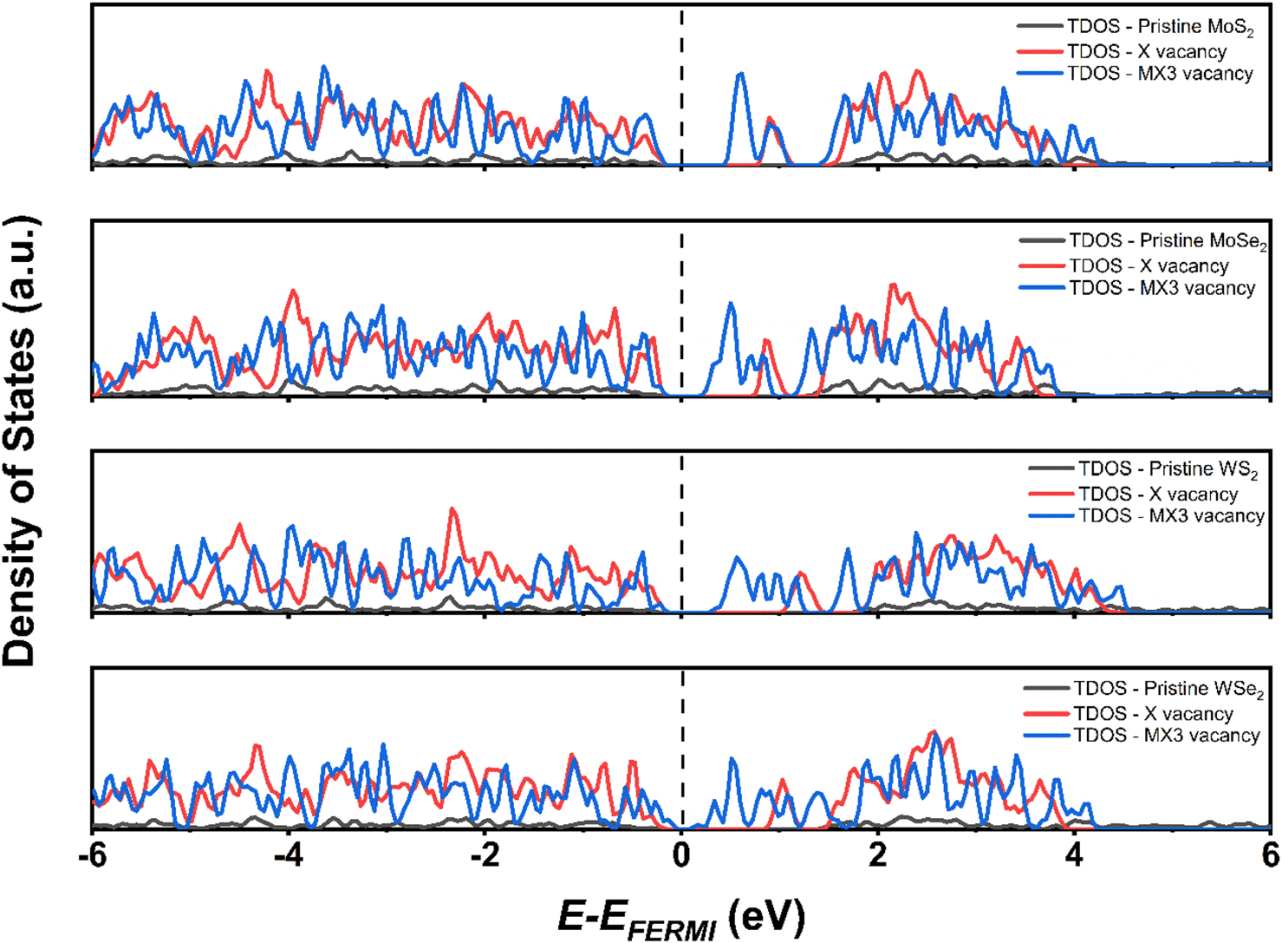
Total DOS of pristine TMDs and defective TMDs with X vacancy and MX3 vacancy defects.

The density of states (DOS) of TMD monolayers with the X and MX3 vacancies were analyzed to understand their influence of the on the electronic properties. As shown in Fig. 6, the DOS images indicate that the TMDs with X vacancy behave as semiconductors. Conversely, the MX3 defective TMDs tend to exhibit metallic behavior. It was already suggested that all 1H TMD monolayers are semiconductors and the band gap energies of MoS_2_, MoSe_2_, WS_2_, and WSe_2_ in their 1H structural polytypes are 1.75, 1.51, 1.85, and 1.64 eV, respectively, which align well with experimental values.^38,41^ Although the DFT-D3 calculations may underestimate the band gap energy of semiconductors,^42^ our results conforms with the reported values at the same functional level,^43^ revealing that the band gaps increase by the increase of the atomic number of TM cations and decreases by the increase of the X anions, while such variation can be ascribed the electronegativity of atoms. According to the definition and trend of the atomic electronegativity in the periodic table, the metal atoms become more active with the increase of the atomic number, while the non-metal atoms have the opposite trend. Consequently, the larger M with the smaller X has the stronger bonding strength in TMD, as suggested by the –pCOHP analysis, which leads to the large band gap energies.

The obtained results in this study imply that the band gap energies reduce with the presence of the vacancy defects on the structure of the TMDs due to the formation of the defect state between the valence band maximum (VBM) and conduction band minimum (CBM). The introduction of the X and MX3 vacancies has huge impact on the electronic and optical properties of TMD monolayers. Following the injection of the X vacancies, the defect state is relatively near to the CBM. Additionally, the MX3 causes a quite large defect condition between the VBM and CBM of pristine TMD. This is because extra dangling bonds form when the unit cell loses four atoms. The MX3 vacancy defect causes the TMD to have lower band gap energies as a result.

**Fig. 7:**
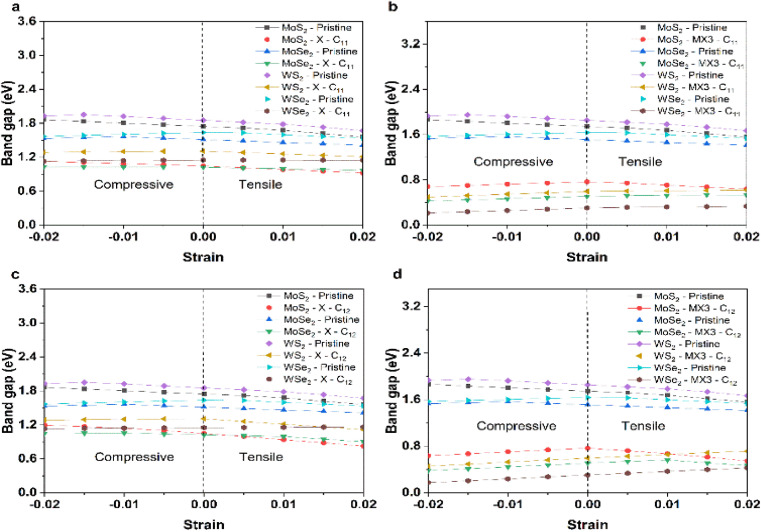
Band gap energies of (a) pristine and TMDs with X vacancies under the compression and tension along the *x* uniaxial direction; (b) pristine and TMDs with MX3 vacancies under the compression and tension along the *x* uniaxial direction; (c) pristine and TMDs with X vacancies under the compression and tension along the *xy* biaxial direction; and (d) pristine and TMDs with MX3 vacancies under the compression and tension along the *xy* biaxial direction.

Fig. 7 shows the change of the band gap energies of the TMD monolayer without or with X and MX3 vacancies under the compression or tension. The band gap energies of pristine TMD and TMDs with the X vacancies generally reduced when the lattice constants become larger. The reduced band gap energies can be explained given the increased M–X bond length leads to the weak interaction between M–X in the pristine TMD monolayer. The similar trend of the band gap energy change supports that the X vacancies have slight effects on the electronic properties of the TMDs. Interestingly, the band gap energies of most TMDs with the MX3 vacancies decrease when they are under higher compression, which could be ascribed to the higher dangling bonding states under the higher compression. Our results suggest that the X and metal complex vacancies may have different influence on the change of the electronic properties, *e.g.*, band gap energies, of defective TMD monolayers under strain. Again, the metal complex vacancies has a relatively larger effect.

## 4. Conclusions

In summary, the first-principles DFT calculations were conducted to investigate the mechanical and electronic properties of defective TMD monolayers. Six different point vacancy defects including X, 2X, 2Xs, M, MX3 and MX6, were considered and compared to that of pristine 2D monolayer systems. The calculated elastic constants, Young's, bulk, and shear moduli, Poison's ratio, and *B*/*G* of the TMD monolayers suggest that the presence of the vacancy defects reduces the mechanical properties of the monolayer systems. However, the impact of the defects on the structural stability of the TMDs is significantly dependent on their types of the vacancies. The X, 2X and 2Xs vacancies have the small influence on the mechanical properties. As a comparison, the introduction of the M and M complex vacancies can significantly alter the mechanical properties. The formation of M and MX6 can even lead some TMDs to be mechanically soft, ductile, and unstable. The formation of MX3 can lead to the least change of the mechanical properties among all the M and M complex vacancies. The further analysis on X and MX3 vacancies reveals that the formation of MX3 has the largest impact on the bonding strength of the atoms next to the vacancy. It explains the larger impact of metal complex vacancies on their mechanical properties. In addition, the formation of MX3 vacancies also have bigger influence on the electronic properties of TMDs. Our comparative study also reveals that the diselenides are more mechanically instable after the formation of M and M complex vacancies. This is because M–Se has the weaker bonding strength based on the COHP analysis results. The findings of this study, therefore, offer a theoretical guidance to manipulate the mechanical and electronic properties of TMD monolayers by tuning their defect.

## Conflicts of interest

There are no conflicts to declare.

## Supplementary Material

RA-013-D3RA00205E-s001
