# Supplementary material for: Vacancy impacts on electronic and mechanical properties of MX2 (M = Mo, W and X = S, Se) monolayers
Source: RSC Adv. 2023 Feb 24;13(10):6498–506. doi: 10.1039/d3ra00205e (PMC9951067; doi:10.1039/d3ra00205e)
Supplement: RA-013-D3RA00205E-s001 [file RA-013-D3RA00205E-s001.pdf]

Electronic Supplementary Material (ESI) for RSC Advances.
This journal is © The Royal Society of Chemistry 2023

Supplementary Information

Vacancy Impacts on electronic and mechanical properties of MX_2 (M = Mo, W and X = S, Se) monolayers

Seyedeh Alieh Kazemi[1]; Sadegh Imani Yengejeh[1]; Samuel Akinlolu Ogunkunle[1]; Lei Zhang[1]; William Wen[1]; Alan Wee-Chung Liew[2]; Yun Wang[1,]*

1. Centre for Catalysis and Clean Energy, School of Environment and Science, Griffith University, Gold Coast Campus, QLD 4222, Australia
2. School of Information and Communication Technology, Griffith University, Gold Coast, Queensland 4215, Australia

[*] Corresponding author email: yun.wang@griffith.edu.au

Lattice vectors and the corresponding cartesian coordinate of MX_2 (M represents a transition metal atoms Molybdenum (Mo) or Tungsten (W), and X represents a chalcogen atom, e.g., Sulphur (S) or Selenium (Se)) with different point vacancy defects:

MoS_2

S Vacancy

Lattice Vectors

9.4111732387357581	-0.0000000000040924	0.0000000000000000
-4.7055866192227152	8.1503151041249655	0.0000000000000000
0.0000000000000000	0.0000000000000000	20.0000000000000000

Cartesian Coordinate

0.0215220167281329	1.8236069506406347	2.4970840362428239	Mo1
-1.5685288730419453	4.5286186393060612	2.5306437196032041	Mo2
-3.1364812075237172	7.2443917465463477	2.5306437196032041	Mo3
3.1370577462783928	1.8594130950773646	2.4638594604674320	Mo4
1.6102989122457840	4.5038369665962072	2.4638594604674320	Mo5
-0.0005765384633660	7.2443917465449843	2.5306437196032041	Mo6
6.2525934758273092	1.8236069506379251	2.4970840362428239	Mo7
4.6638165804051335	4.5038369665948794	2.4638594604674320	Mo8
3.1370577463738334	7.2198731269898326	2.4970840362428239	Mo9
1.6001919929660162	0.9704986262481915	4.0744399626004215	S1
0.0720435488763544	3.6173293729886216	4.0744399626004215	S2
-1.5685289906493578	6.3391339472342754	4.1132287304178297	S3
4.6739232642797202	0.9704986262468549	4.0744399626004215	S4
1.6089091845806180	6.2792587383396787	4.0744399626004215	S5
7.8426442479895924	0.9055905444815892	4.0596985465995781	S6
6.2020717084636665	3.6173293729859561	4.0744399626004215	S7
4.6652060728542262	6.2792587383383500	4.0744399626004215	S8
1.5912787854617365	0.8969430950584872	0.9256009901253039	S9
0.0038859865259409	3.6463880744567678	0.9256009901253039	S10
-1.5685289906493578	6.3391339472342754	0.9689671040588620	S11
4.6828364717813793	0.8969430950571429	0.9256009901253039	S12
3.1370576286700667	3.6223622458579610	0.8797783937917820	S13
1.5496648297334481	6.3237555680612960	0.9256009901253039	S14
7.8426442479895924	0.9055905444815892	0.9366893825510659	S15
6.2702292708151166	3.6463880744540429	0.9256009901253039	S16
4.7244504277029806	6.3237555680599149	0.9256009901253039	S17

2S Vacancy

Lattice Vectors

9.3153270716903354	-0.0000000000057800	0.0000000000000000
-4.6576635357029819	8.0673098886103141	0.0000000000000000
0.0000000000000000	0.0000000000000000	20.0000000000000000

Cartesian Coordinate

0.0202885694591086	1.8044492153240670	2.4617752022867019	Mo1
-1.5525545118684363	4.5369699026789050	2.5775729523761020	Mo2
-3.0573641753539080	7.1433766956248128	2.5775729523761020	Mo3
3.1051090239287853	1.8166203503318090	2.4647365043725240	Mo4
1.5732393088520866	4.4698965274903877	2.4647365043725240	Mo5
-0.0477448482910221	7.1433766956229450	2.5775729523761020	Mo6
6.1899294783980325	1.8044492153202389	2.4617752022867019	Mo7
4.6369787390989448	4.4698965274884870	2.4647365043725240	Mo8
3.1051090240227426	7.1475149746662634	2.4617752022867019	Mo9

1.5643211510696766	0.9476288129229042	4.0841495073749465	S1
0.0502766501811220	3.5700308134510088	4.0841495073749465	S2
-1.5525546282793603	6.2745743353975199	4.1832329121015741	S3
4.6458966638740993	0.9476288129209921	4.0841495073749465	S4
1.5910644066303687	6.2387534912063991	4.0841495073749465	S5
7.7627724433161527	0.8963677429868090	4.0177063228830443	S6
6.1599411648550939	3.5700308134472176	4.0841495073749465	S7
4.6191534084999182	6.2387534912045206	4.0841495073749465	S8
1.6164002707728709	0.8182842160326876	0.9195931800152840	S9
-0.0356994967138822	3.6798049525676175	0.9195931800152840	S10
4.5938175441662787	0.8182842160308402	0.9195931800152840	S11
3.1051089075183467	3.5854710391921927	0.8841625736687320	S12
1.4530091400282350	6.2583239489801139	0.9195931800152840	S13
7.7627724433161527	0.8963677429868090	0.8801890498989461	S14
6.2459173117539661	3.6798049525637202	0.9195931800152840	S15
4.7572086751026754	6.2583239489780631	0.9195931800152840	S16

2Ss Vacancy

Lattice Vectors

9.3117059180975463	-0.0000000000059918	0.0000000000000000
-4.6558529589068254	8.0641738776081713	0.0000000000000000
0.0000000000000000	0.0000000000000000	20.0000000000000000

Cartesian Coordinate

0.0286195435519880	1.8085622140967530	2.5000000000000000	Mo1
-1.5519509862706384	4.4859038497842230	2.5000000000000000	Mo2
-3.0988728092124260	7.1652510425267879	2.5000000000000000	Mo3
3.1039019727335395	1.9495119841354340	2.5000000000000000	Mo4
1.6883268397005033	4.4013600365440597	2.5000000000000000	Mo5
-0.0050291632344663	7.1652510425247948	2.5000000000000000	Mo6
6.1791844019100353	1.8085622140927808	2.5000000000000000	Mo7
4.5194771058527667	4.4013600365422061	2.5000000000000000	Mo8
3.1039019728247563	7.1351076290321087	2.5000000000000000	Mo9
1.6105271796682255	0.9645631302668081	4.0759138736264564	S1
0.0886487391102793	3.6005339123260445	4.0759138736264537	S2
-1.5519511026354826	6.2721352157378503	4.0805337971141560	S3
4.5972765329714544	0.9645631302648725	4.0759138736264537	S4
1.5820234158074125	6.1871347270104904	4.0759138736264564	S5
7.7597548153673994	0.8960192973284270	4.0707216820823460	S6
6.1191549736221891	3.6005339123221440	4.0759138736264564	S7
4.6257802970160942	6.1871347270085284	4.0759138736264537	S8
1.6105271796682255	0.9645631302668081	0.9240862463735540	S9
0.0886487391102793	3.6005339123260445	0.9240862463735540	S10
-1.5519511026354826	6.2721352157378503	0.9194663228858541	S11
4.5972765329714544	0.9645631302648725	0.9240862463735540	S12
1.5820234158074125	6.1871347270104904	0.9240862463735540	S13
7.7597548153673994	0.8960192973284270	0.9292784379176640	S14
6.1191549736221891	3.6005339123221440	0.9240862463735540	S15
4.6257802970160942	6.1871347270085284	0.9240862463735540	S16

Mo Vacancy

Lattice Vectors

9.5202377734888675	-0.0000000000021780	0.0000000000000000	
-4.7601188865958894	8.2447677618707615	0.0000000000000000	
0.0000000000000000	0.0000000000000000	20.0000000000000000	

Cartesian Coordinate

-0.0101326416111291	1.8693725632041622	2.5000000000000000	Mo1
-1.5595548480780319	4.5530505472514307	2.5000000000000000	Mo2
-3.1734125910309059	7.3286825301149658	2.5000000000000000	Mo3
3.1835452328414684	1.8693725632034317	2.5000000000000000	Mo4
0.0372840891981321	7.3188567180178632	2.5000000000000000	Mo5
6.3468251823588986	1.8321706888663589	2.5000000000000000	Mo6
4.7329674394050052	4.5530505472499909	2.5000000000000000	Mo7
3.1361285022285719	7.3188567180171544	2.5000000000000000	Mo8
1.5867061765953216	0.9323505132143106	4.0592076640813000	S1
-0.0345795865066338	3.6443767396860336	4.0671551459199398	S2
-1.5726203127466660	6.4044645105978573	4.0592076640813000	S3
4.7632368919755708	0.9178855338275690	4.0564750955455500	S4
3.2079919397949332	3.6443767396852915	4.0671551459199398	S5
1.5867061766947437	6.4525260550373140	4.0671551459199398	S6
7.9304132347033525	0.9178855338268443	4.0564750955455500	S7
6.3468250633888790	3.6607407048812854	4.0564750955455500	S8
4.7460326661344903	6.4044645105964113	4.0592076640813000	S9
1.5867061765953216	0.9323505132143106	0.9407924559187100	S10
-0.0345795865066338	3.6443767396860336	0.9328449740800699	S11
-1.5726203127466660	6.4044645105978573	0.9407924559187100	S12
4.7632368919755708	0.9178855338275690	0.9435250244544601	S13
3.2079919397949332	3.6443767396852915	0.9328449740800699	S14
1.5867061766947437	6.4525260550373140	0.9328449740800699	S15
7.9304132347033525	0.9178855338268443	0.9435250244544601	S16
6.3468250633888790	3.6607407048812854	0.9435250244544601	S17
4.7460326661344903	6.4044645105964113	0.9407924559187100	S18

MoS3 Vacancy

Lattice Vectors

9.4603592762838016	-0.0000000000034214	0.0000000000000000
-4.7301796379953798	8.1929114621522015	0.0000000000000000
0.0000000000000000	0.0000000000000000	20.0000000000000000

Cartesian Coordinate

-0.0114642851495199	1.7768709459257446	2.4168612673825858	Mo1
-1.6203699208239233	4.5635772515713331	2.4168612673825858	Mo2
-3.1534530919643489	7.2825880410036392	2.5424831387039140	Mo3
3.1649173773076975	1.7768709459245957	2.4168612673825858	Mo4
-0.0321790895460854	7.3144044633605887	2.4168612673825858	Mo5
6.3069061842217389	1.8206470662332435	2.4447954852588798	Mo6
4.7738230130817074	4.5635772515690203	2.4168612673825858	Mo7
3.1856321819023279	7.3144044633594243	2.4168612673825858	Mo8
1.5767264278118480	1.0635150130741815	4.0704694338101879	S1
-1.4440588988704697	6.2956686777847306	4.0704694338101879	S2
4.7373111918935455	0.9144409457778009	4.0153622900398043	S3
7.8765009400085839	0.9144409457766074	4.0153622900398043	S4
6.3069060659997014	3.6330590149314852	4.0153622900398043	S5
4.5975117546813742	6.2956686777825457	4.0704694338101879	S6

1.5767264278081461	0.8546734493933973	0.9089098369297239	S7
0.1555366980254966	3.7310931831290590	1.2670584157623921	S8
-1.6249209983830393	6.4000894596251587	0.9089098369297239	S9
4.7069432734445815	0.8969080198867991	0.8420867500848319	S10
2.9979161576935436	3.7310931831280310	1.2670584157623921	S11
1.5767264279035427	6.1926660023844269	1.2670584157623921	S12
7.9068688584568889	0.8969080198856418	0.8420867500848319	S13
6.3069060660002947	3.6681248667134518	0.8420867500848319	S14
4.7783738541976781	6.4000894596228433	0.9089098369297239	S15

MoS6 Vacancy

Lattice Vectors

9.0409642721753407	-0.0000000000106892	0.0000000000000000
-4.5204821359540679	7.8297047343828554	0.0000000000000000
0.0000000000000000	0.0000000000000000	20.0000000000000000

Cartesian Coordinate

-0.0964879062251843	1.8498663886355802	2.5000000000000000	Mo1
-1.4598674860560492	4.2113090909657496	2.5000000000000000	Mo2
-3.0136547572730126	6.9597376130097528	2.5000000000000000	Mo3
3.1101426636801159	1.8498663886317890	2.5000000000000000	Mo4
0.1434477989440506	6.9883326250415099	2.5000000000000000	Mo5
6.0273095148132283	1.7399344567474080	2.5000000000000000	Mo6
4.4735222435914750	4.2113090909586788	2.5000000000000000	Mo7
2.8702069586862420	6.9883326250382858	2.5000000000000000	Mo8
1.5068272657023301	1.0808869368014453	4.0628856988341520	S1
-1.3241656162240014	5.9843104442907764	4.0628856988341520	S2
4.5005518482344016	0.8584604791361425	4.1344913545268023	S3
7.5540669553378788	0.8584604791325324	4.1344913545268023	S4
6.0273094018312641	3.5028821327140856	4.1344913545268023	S5
4.3378201477959042	5.9843104442840263	4.0628856988341520	S6
1.5068272657023301	1.0808869368014453	0.9371144211658580	S7
-1.3241656162240014	5.9843104442907764	0.9371144211658580	S8
4.5005518482344016	0.8584604791361425	0.8655087654732080	S9
7.5540669553378788	0.8584604791325324	0.8655087654732080	S10
6.0273094018312641	3.5028821327140856	0.8655087654732080	S11
4.3378201477959042	5.9843104442840263	0.9371144211658580	S12

$MoSe_2$

Se Vacancy

Lattice Vectors

9.7777041247952035	0.0000000000017589	0.0000000000000000
-4.8888520622415781	8.4677401627168063	0.0000000000000000
0.0000000000000000	0.0000000000000000	20.0000000000000000

Cartesian Coordinate

0.0275129288034457	1.8976047101406495	2.5046763361216060	Mo1
-1.6296173540458823	4.7033794201618164	2.5387511680938242	Mo2
-3.2600320987540998	7.5273405954814301	2.5387511680938242	Mo3
3.2592347083013662	1.9631209837086792	2.4590778750231839	Mo4
1.7001125758737556	4.6635997323505487	2.4590778750231839	Mo5
0.0007973907664720	7.5273405954820163	2.5387511680938242	Mo6
6.4909564877965611	1.8976047101417519	2.5046763361216060	Mo7
4.8183568408281463	4.6635997323509892	2.4590778750231839	Mo8
3.2592347084031239	7.4951110281278162	2.5046763361216060	Mo9
1.6733104422512044	1.0168474407462100	4.1929768650254582	Se1
0.0876535417762150	3.7632857558145449	4.1929768650254582	Se2
-1.6296174762324536	6.5860201030363807	4.2345584650107080	Se3
4.8451587298738605	1.0168474407467807	4.1929768650254582	Se4
1.6735776856381563	6.5101869498323204	4.1929768650254582	Se5
8.1480866484587011	0.9408599945597027	4.1765090526304496	Se6
6.4308156304499562	3.7632857558156254	4.1929768650254582	Se7
4.8448914866892405	6.5101869498328311	4.1929768650254582	Se8
1.6539645406134653	0.9343744938316610	0.8215252051438420	Se9
0.0065569238052330	3.7877681869958066	0.8215252051438420	Se10
-1.6296174762324536	6.5860201030363807	0.8652566425911340	Se11
4.8645046315084919	0.9343744938322386	0.8215252051438420	Se12
3.2592345861130707	3.7634400487980719	0.7248353601465860	Se13
1.6118269693039773	6.5681774655655190	0.8215252051438420	Se14
8.1480866484587011	0.9408599945597027	0.8376814436019241	Se15
6.5119122484218757	3.7877681869969773	0.8215252051438420	Se16
4.9066422030255934	6.5681774655661114	0.8215252051438420	Se17

2Se Vacancy

Lattice Vectors

9.6312591405524426	-0.0000000000010161	0.0000000000000000
-4.8156295701249148	8.3409150861090851	0.0000000000000000
0.0000000000000000	0.0000000000000000	20.0000000000000000

Cartesian Coordinate

0.0249774621457253	1.8679575062707414	2.4568105672175018	Mo1
-1.6052098566729394	4.7248275704500591	2.5915289173255478	Mo2
-3.1316237165634679	7.3686539292279178	2.5915289173255478	Mo3
3.2104197135520867	1.9063515316897588	2.4545582040995839	Mo4
1.6509487892650530	4.6074344055530796	2.4545582040995839	Mo5
-0.0787959966866971	7.3686539292274773	2.5915289173255478	Mo6
6.3958619649570529	1.8679575062700693	2.4568105672175018	Mo7
4.7698906379370127	4.6074344055526915	2.4545582040995839	Mo8
3.2104197136514410	7.3853053302546012	2.4568105672175018	Mo9
1.6265883722948780	0.9872219078977691	4.1963963255946615	Se1

0.0630435407328878	3.6953609961468277	4.1963963255946615	Se2
-1.6052099770317674	6.4873783771376070	4.3538666278539040	Se3
4.7942508139944486	0.9872219078973757	4.1963963255946615	Se4
1.6468747616323856	6.4386371412589050	4.1963963255946615	Se5
8.0260491634197706	0.9267683197308924	4.1271066473295539	Se6
6.3577956456546580	3.6953609961461638	4.1963963255946615	Se7
4.7739644248546185	6.4386371412585746	4.1963963255946615	Se8
1.6748397758851745	0.8327546717019294	0.8083190354557240	Se9
-0.0466033080670241	3.8143815555244309	0.8083190354557240	Se10
4.7459994103984791	0.8327546717015462	0.8083190354557240	Se11
3.2104195931939503	3.7070733484342795	0.7244811990609880	Se12
1.4889765092378240	6.4740838180771050	0.8083190354557240	Se13
8.0260491634197706	0.9267683197308924	0.7575612535256939	Se14
6.4674424944589557	3.8143815555237435	0.8083190354557240	Se15
4.9318626772504990	6.4740838180768012	0.8083190354557240	Se16

2Ses Vacancy

Lattice Vectors

9.6565062585701451	-0.0000000000006961	0.0000000000000000
-4.8282531291330901	8.3627797316843431	0.0000000000000000
0.0000000000000000	0.0000000000000000	20.0000000000000000

Cartesian Coordinate

0.0276054587199372	1.8743335911272641	2.5000000000000000	Mo1
-1.6094177096771003	4.6552039492660704	2.5000000000000000	Mo2
-3.2108548798969174	7.4289744932898936	2.5000000000000000	Mo3
3.2188354195617261	2.1010921428585694	2.5000000000000000	Mo4
1.8195991055126770	4.5246405306512596	2.5000000000000000	Mo5
-0.0079805393563950	7.4289744932896635	2.5000000000000000	Mo6
6.4100653803950332	1.8743335911267449	2.5000000000000000	Mo7
4.6180717336983843	4.5246405306508199	2.5000000000000000	Mo8
3.2188354196578866	7.4017060219065209	2.5000000000000000	Mo9
1.6797488157096274	1.0109324885344613	4.1831415767342159	Se1
0.1059498745026883	3.7368322156754821	4.1831415767342159	Se2
-1.6094178303497810	6.5043842125243660	4.1998110985097981	Se3
4.7579217819612847	1.0109324885342394	4.1831415767342159	Se4
1.6450363576769629	6.4026082016786816	4.1831415767342159	Se5
8.0470884281190092	0.9291977247343592	4.1887613381167901	Se6
6.3317207232672708	3.7368322156749736	4.1831415767342159	Se7
4.7926342401897841	6.4026082016784551	4.1831415767342159	Se8
1.6797488157096274	1.0109324885344613	0.8168585432657940	Se9
0.1059498745026883	3.7368322156754821	0.8168585432657940	Se10
-1.6094178303497810	6.5043842125243660	0.8001890214902120	Se11
4.7579217819612847	1.0109324885342394	0.8168585432657940	Se12
1.6450363576769629	6.4026082016786816	0.8168585432657940	Se13
8.0470884281190092	0.9291977247343592	0.8112387818832180	Se14
6.3317207232672708	3.7368322156749736	0.8168585432657940	Se15
4.7926342401897841	6.4026082016784551	0.8168585432657940	Se16

Mo Vacancy

Lattice Vectors

9.8830278531224884	0.0000000000037801	0.0000000000000000

-4.9415139264018082	8.5589531870671127	0.0000000000000000
0.0000000000000000	0.0000000000000000	20.0000000000000000

Cartesian Coordinate

-0.0414319799453589	1.8537017125598958	2.5000000000000000	Mo1
-1.7097059010192157	4.7432369048796188	2.5000000000000000	Mo2
-3.2943426175657637	7.6079584664860729	2.5000000000000000	Mo3
3.3357745977219397	1.8537017125611877	2.5000000000000000	Mo4
-0.0211026121310489	7.6679835949528643	2.5000000000000000	Mo5
6.5886852354503995	1.9019896751105383	2.5000000000000000	Mo6
5.0040485189033790	4.7432369048821865	2.5000000000000000	Mo7
3.3154452301242472	7.6679835949541406	2.5000000000000000	Mo8
1.6471711853339861	0.9680026779621058	4.1740907756626200	Se1
0.0225158242193357	3.8169787589356314	4.0787936455478766	Se2
-1.6324421560545890	6.6484596145805037	4.1740907756626200	Se3
4.9687556677973124	0.9667228727402746	4.2266693008981795	Se4
3.2718265465547072	3.8169787589367528	4.0787936455478766	Se5
1.6471711854393352	6.6309643892532382	4.0787936455478766	Se6
8.2086145559929236	0.9667228727414530	4.2266693008981795	Se7
6.5886851119473668	3.7725229745805628	4.2266693008981795	Se8
4.9267845269341564	6.6484596145829515	4.1740907756626200	Se9
1.6471711853339861	0.9680026779621058	0.8259093443373899	Se10
0.0225158242193357	3.8169787589356314	0.9212064744521340	Se11
-1.6324421560545890	6.6484596145805037	0.8259093443373899	Se12
4.9687556677973124	0.9667228727402746	0.7733308191018300	Se13
3.2718265465547072	3.8169787589367528	0.9212064744521340	Se14
1.6471711854393352	6.6309643892532382	0.9212064744521340	Se15
8.2086145559929236	0.9667228727414530	0.7733308191018300	Se16
6.5886851119473668	3.7725229745805628	0.7733308191018300	Se17
4.9267845269341564	6.6484596145829515	0.8259093443373899	Se18

MoSe3 Vacancy

Lattice Vectors

9.8096604664089249	0.0000000000001946	0.0000000000000000
-4.9048302330492879	8.4954151663676800	0.0000000000000000
0.0000000000000000	0.0000000000000000	20.0000000000000000

Cartesian Coordinate

-0.0216558171914039	1.8307075014549963	2.4338276537071120	Mo1
-1.6952755945283831	4.7295019884320135	2.4338276537071120	Mo2
-3.2698868219983641	7.5514802252850544	2.5608793025114100	Mo3
3.2915426393945433	1.8307075014550620	2.4338276537071120	Mo4
-0.0386763661829645	7.5988160196005374	2.4338276537071120	Mo5
6.5397736443070773	1.8878701143734182	2.4693131225379261	Mo6
4.9651624168374546	4.7295019884322063	2.4338276537071120	Mo7
3.3085631885969260	7.5988160196006644	2.4338276537071120	Mo8
1.6349432884678838	1.1185844638987690	4.2050694720073523	Se1
-1.4836926566472890	6.5202203712927949	4.2050694720073523	Se2
4.9117442589607778	0.9479268803037438	4.1627703506284783	Se3
8.1678027843775407	0.9479268803038085	4.1627703506284783	Se4
6.5397735217206652	3.7677562795095660	4.1627703506284783	Se5
4.7535792337803855	6.5202203712929183	4.2050694720073523	Se6
1.6349432884634973	0.8803378684196494	0.8071251708595640	Se7

0.1475898184436888	3.8609511425050100	1.1448897898961261	Se8
-1.6900202606962642	6.6393436690323817	0.8071251708595640	Se9
4.8948258231169657	0.9381590168151621	0.7357945702243280	Se10
3.1222967585920873	3.8609511425050087	1.1448897898961261	Se11
1.6349432885649264	6.4371229214745842	1.1448897898961261	Se12
8.1847212202210660	0.9381590168152274	0.7357945702243280	Se13
6.5397735217209876	3.7872920064866684	0.7357945702243280	Se14
4.9599068378337119	6.6393436690325132	0.8071251708595640	Se15

MoSe6 Vacancy

Lattice Vectors

9.2349513896829247	-0.0000000000088104	0.0000000000000000
-4.6174756947031685	7.9977025061486797	0.0000000000000000
0.0000000000000000	0.0000000000000000	20.0000000000000000

Cartesian Coordinate

-0.1943322796041949	1.9368106007535537	2.5000000000000000	Mo1
-1.4981561501673171	4.1950997887487196	2.5000000000000000	Mo2
-3.0783171297713698	7.1090689672213401	2.5000000000000000	Mo3
3.2726494095656378	1.9368106007502461	2.5000000000000000	Mo4
0.2353346944695511	7.1975940060102230	2.5000000000000000	Mo5
6.1566342598193273	1.7772672964496989	2.5000000000000000	Mo6
4.5764732802065637	4.1950997887428674	2.5000000000000000	Mo7
2.8429824356736315	7.1975940060076216	2.5000000000000000	Mo8
1.5391584495313650	1.2137440794782532	4.1825963637140262	Se1
-1.2576047369863050	6.0578800153911994	4.1825963637140262	Se2
4.6185044589125779	0.8892276134255659	4.3197248246787021	Se3
7.6947638298216257	0.8892276134226877	4.3197248246787021	Se4
6.1566341444132178	3.5533463772494023	4.3197248246787021	Se5
4.3359216362162671	6.0578800153856927	4.1825963637140262	Se6
1.5391584495313650	1.2137440794782532	0.8174037562859839	Se7
-1.2576047369863050	6.0578800153911994	0.8174037562859839	Se8
4.6185044589125779	0.8892276134255659	0.6802752953213080	Se9
7.6947638298216257	0.8892276134226877	0.6802752953213080	Se10
6.1566341444132178	3.5533463772494023	0.6802752953213080	Se11
4.3359216362162671	6.0578800153856927	0.8174037562859839	Se12

WS_2

S Vacancy

Lattice Vectors

9.4267623983714870	-0.0000000000038032	0.0000000000000000
-4.7133811990400813	8.1638157123928057	0.0000000000000000
0.0000000000000000	0.0000000000000000	20.0000000000000000

Cartesian Coordinate

0.0197631224100241	1.8255915878262192	2.5012425979849700	W1
-1.5711270663143118	4.5367677192726266	2.5335921733389459	W2
-3.1411157670879923	7.2560679163934632	2.5335921733389459	W3
3.1422541328241187	1.8831633495419393	2.4601134640724358	W4
1.6308672358520582	4.5009622450605082	2.4601134640724358	W5
-0.0011383654435280	7.2560679163921966	2.5335921733389459	W6
6.2647451432360919	1.8255915878236997	2.5012425979849700	W7
4.6536410298895268	4.5009622450592888	2.4601134640724358	W8
3.1422541329195548	7.2339046640117601	2.5012425979849700	W9
1.6063946019088733	0.9733026620689033	4.0833252183234938	S1
0.0749750262643092	3.6257991748608660	4.0833252183234938	S2
-1.5711271841164565	6.3496344202940467	4.1259118692995864	S3
4.6781134280378494	0.9733026620676640	4.0833252183234938	S4
1.6108344393762608	6.2859857115582365	4.0833252183234938	S5
7.8556352141578891	0.9070906120296600	4.0773569050473659	S6
6.2095330037770680	3.6257991748583911	4.0833252183234938	S7
4.6736735907600426	6.2859857115570001	4.0833252183234938	S8
1.5924461953629012	0.8994345687691012	0.9205387886510159	S9
0.0040291776653354	3.6506535471009314	0.9205387886510159	S10
-1.5711271841164565	6.3496344202940467	0.9607235885545160	S11
4.6920618345811178	0.8994345687678507	0.9205387886510159	S12
3.1422540150206664	3.6283625161618822	0.8322039509603479	S13
1.5538369973223389	6.3349994326180887	0.9205387886510159	S14
7.8556352141578891	0.9070906120296600	0.9291444608143700	S15
6.2804788523768611	3.6506535470983987	0.9205387886510159	S16
4.7306710328156480	6.3349994326168071	0.9205387886510159	S17

2S Vacancy

Lattice Vectors

9.3228680414213638	-0.0000000000056772	0.0000000000000000
-4.6614340205682865	8.0738405599664240	0.0000000000000000
0.0000000000000000	0.0000000000000000	20.0000000000000000

Cartesian Coordinate

0.0181522827068092	1.8046670899534245	2.4627066587017059	W1
-1.5538113401564624	4.5611108568260459	2.5825136082796978	W2
-3.0421132231405501	7.1389253352226305	2.5825136082796978	W3
3.1076226805063119	1.8409320100824900	2.4583567874697101	W4
1.5942938237528685	4.4620944786084307	2.4583567874697101	W5
-0.0655094570813803	7.1389253352208177	2.5825136082796978	W6
6.1970930783044009	1.8046670899496617	2.4627066587017059	W7
4.6209515373521119	4.4620944786065291	2.4583567874697101	W8
3.1076226805999809	7.1557867873943071	2.4627066587017059	W9
1.5700227257792250	0.9492554519239217	4.0897230104506841	S1

0.0532793199137700	3.5763320929969611	4.0897230104506841	S2
-1.5538114566619639	6.2796537464341542	4.2316474243429516	S3
4.6452224021301909	0.9492554519220491	4.0897230104506841	S4
1.5908791581362558	6.2395331344132536	4.0897230104506841	S5
7.7690565846644377	0.8970933731194385	4.0360776126404119	S6
6.1619658080882784	3.5763320929931837	4.0897230104506841	S7
4.6243659699597650	6.2395331344114071	4.0897230104506841	S8
1.6162687828400086	0.8179632031517853	0.9142495896139741	S9
-0.0373000743099743	3.6820284776243923	0.9142495896139741	S10
4.5989763450647114	0.8179632031499688	0.9142495896139741	S11
3.1076225640011867	3.5883735597768247	0.8288962856946119	S12
1.4540537068479340	6.2651289985580689	0.9142495896139741	S13
7.7690565846644377	0.8970933731194385	0.8688128735799699	S14
6.2525452023157175	3.6820284776205621	0.9142495896139741	S15
4.7611914212489239	6.2651289985560554	0.9142495896139741	S16

2Ss Vacancy

Lattice Vectors

9.3349520522472584	-0.0000000000055554	0.0000000000000000
-4.6674760259809371	8.0843056203210839	0.0000000000000000

Cartesian Coordinate

0.0298589029742578	1.8137514793790162	2.5000000000000000	W1
-1.5558253419619017	4.4919731154151092	2.5000000000000000	W2
-3.1110512717640511	7.1857034431516462	2.5000000000000000	W3
3.1116506841170839	1.9607723173589895	2.5000000000000000	W4
1.6980785741685951	4.4091510320163305	2.5000000000000000	W5
-0.0005994120646955	7.1857034431497375	2.5000000000000000	W6
6.1934424652546527	1.8137514793753482	2.5000000000000000	W7
4.5252227941518353	4.4091510320145906	2.5000000000000000	W8
3.1116506842086373	7.1515714226354872	2.5000000000000000	W9
1.6119590077255677	0.9631481713533422	4.0853059448527063	S1
0.0842649069863688	3.6091919723264105	4.0853059448527063	S2
-1.5558254586170990	6.2877932377920684	4.0862229676764619	S3
4.6113421270994621	0.9631481713515572	4.0853059448527063	S4
1.5839564667191639	6.2067339493734757	4.0853059448527063	S5
7.7791265935349987	0.8982561575743282	4.0800290553069383	S6
6.1390362279320687	3.6091919723228072	4.0853059448527063	S7
4.6393446682909687	6.2067339493716567	4.0853059448527063	S8
1.6119590077255677	0.9631481713533422	0.9146941751473039	S9
0.0842649069863688	3.6091919723264105	0.9146941751473039	S10
-1.5558254586170990	6.2877932377920684	0.9137771523235481	S11
4.6113421270994621	0.9631481713515572	0.9146941751473039	S12
1.5839564667191639	6.2067339493734757	0.9146941751473039	S13
7.7791265935349987	0.8982561575743282	0.9199710646930720	S14
6.1390362279320687	3.6091919723228072	0.9146941751473039	S15
4.6393446682909687	6.2067339493716567	0.9146941751473039	S16

W Vacancy

Lattice Vectors

9.5741194205013915	-0.0000000000012311	0.0000000000000000
-4.7870597101004790	8.2914306369802002	0.0000000000000000

0.0000000000000000	0.0000000000000000	20.0000000000000000	

Cartesian Coordinate

-0.0245987785143460	1.8836532286752714	2.5000000000000000	W1
-1.5723810468204893	4.5644907565069222	2.5000000000000000	W2
-3.1913731400335936	7.3701606417486119	2.5000000000000000	W3
3.2159719187497524	1.8836532286748549	2.5000000000000000	W4
0.0479043018624381	7.3709073030830838	2.5000000000000000	W5
6.3827462803676198	1.8425402170943437	2.5000000000000000	W6
4.7637541871529656	4.5644907565061077	2.5000000000000000	W7
3.1434688385717946	7.3709073030826859	2.5000000000000000	W8
1.5956864504242092	0.9448583579155611	4.0533365730905766	S1
-0.0416153650708155	3.6610536869013197	4.0738514558123020	S2
-1.5752586138019957	6.4370963173109850	4.0533365730905766	S3
4.7932830106826856	0.9248631410083337	4.0638505415924442	S4
3.2329882660175846	3.6610536869008987	4.0738514558123020	S5
1.5956864505247625	6.4969436187345551	4.0738514558123020	S6
7.9722093106663996	0.9248631410079249	4.0638505415924442	S7
6.3827461607244196	3.6778940735390262	4.0638505415924442	S8
4.7666315148494203	6.4370963173101696	4.0533365730905766	S9
1.5956864504242092	0.9448583579155611	0.9466635469094340	S10
-0.0416153650708155	3.6610536869013197	0.9261486641877080	S11
-1.5752586138019957	6.4370963173109850	0.9466635469094340	S12
4.7932830106826856	0.9248631410083337	0.9361495784075660	S13
3.2329882660175846	3.6610536869008987	0.9261486641877080	S14
1.5956864505247625	6.4969436187345551	0.9261486641877080	S15
7.9722093106663996	0.9248631410079249	0.9361495784075660	S16
6.3827461607244196	3.6778940735390262	0.9361495784075660	S17
4.7666315148494203	6.4370963173101696	0.9466635469094340	S18

WS3 Vacancy

Lattice Vectors

9.4640322670922874	-0.0000000000033128	0.0000000000000000
-4.7320161333994673	8.1960923655000961	0.0000000000000000
0.0000000000000000	0.0000000000000000	20.0000000000000000

Cartesian Coordinate

-0.0334217312352341	1.7822074736662517	2.4193844349622400	W1
-1.6279514055704232	4.5440138838640394	2.4193844349622400	W2
-3.1546774222337062	7.2854155106752065	2.5298095793618818	W3
3.1880991536631420	1.7822074736651239	2.4193844349622400	W4
-0.0171909630712728	7.3339328089942200	2.4193844349622400	W5
6.3093548447607644	1.8213539336729527	2.4520963819261961	W6
4.7826288280971125	4.5440138838617949	2.4193844349622400	W7
3.1718683856978869	7.3339328089931035	2.4193844349622400	W8
1.5773385929011088	1.0903827885742439	4.0741682177990421	S1
-1.4217089704847385	6.2848855428114456	4.0741682177990421	S2
4.7475100126149012	0.9196223702736160	4.0438919358322778	S3
7.8711994402735606	0.9196223702725226	4.0438919358322778	S4
6.3093547264926428	3.6248167681454304	4.0438919358322778	S5
4.5763861564728767	6.2848855428093460	4.0741682177990421	S6
1.5773385928971397	0.8686069524311306	0.9053579560986701	S7
0.1543637298525975	3.7318297044775988	1.2478399680163941	S8

-1.6137724785291421	6.3957734608830696	0.9053579560986701	S9
4.7161531360372217	0.9015184691408697	0.8293732012792541	S10
3.0003134560442972	3.7318297044766027	1.2478399680163941	S11
1.5773385929925552	6.1964944652407175	1.2478399680163941	S12
7.9025563168505242	0.9015184691397544	0.8293732012792541	S13
6.3093547264932575	3.6610245704110014	0.8293732012792541	S14
4.7684496645212491	6.3957734608808350	0.9053579560986701	S15

WS6 Vacancy

Lattice Vectors

9.0872946098196330	-0.0000000000102040	0.0000000000000000
-4.5436473047750621	7.8698279837480527	0.0000000000000000
0.0000000000000000	0.0000000000000000	20.0000000000000000

Cartesian Coordinate

-0.1307136089836106	1.8410603042513092	2.5000000000000000	W1
-1.5000500764176130	4.2128206385654430	2.5000000000000000	W2
-3.0290982031534193	6.9954027239222150	2.5000000000000000	W3
3.1598118123199574	1.8410603042476141	2.5000000000000000	W4
0.1452126342829985	7.0624992451985200	2.5000000000000000	W5
6.0581964065763465	1.7488507347500633	2.5000000000000000	W6
4.5291482798350202	4.2128206385586173	2.5000000000000000	W7
2.8838855692320644	7.0624992451953892	2.5000000000000000	W8
1.5145489880640977	1.0896222277860954	4.0596730581034981	S1
-1.3281832172146961	6.0133788397690768	4.0596730581034981	S2
4.5331491203367387	0.8683642455453541	4.1639931221207860	S3
7.5832434656034486	0.8683642455419291	4.1639931221207860	S4
6.0581962930153237	3.5098234324723805	4.1639931221207860	S5
4.3572811935115094	6.0133788397626926	4.0596730581034981	S6
1.5145489880640977	1.0896222277860954	0.9403270618965099	S7
-1.3281832172146961	6.0133788397690768	0.9403270618965099	S8
4.5331491203367387	0.8683642455453541	0.8360069978792239	S9
7.5832434656034486	0.8683642455419291	0.8360069978792239	S10
6.0581962930153237	3.5098234324723805	0.8360069978792239	S11
4.3572811935115094	6.0133788397626926	0.9403270618965099	S12

WSe_2

Se Vacancy

Lattice Vectors

9.7644500272290689	0.0000000000014230	0.0000000000000000
-4.8822250134590117	8.4562617775206785	0.0000000000000000
0.0000000000000000	0.0000000000000000	20.0000000000000000

Cartesian Coordinate

0.0197695848084287	1.8905833360303670	2.5084124025336503	W1
-1.6274083377850683	4.7007991467958901	2.5431993187757480	W2
-3.2523261077731456	7.5152392826119838	2.5431993187757480	W3
3.2548166757798787	1.9951839232852400	2.4574617039696980	W4
1.7278798961572610	4.6399160056077866	2.4574617039696980	W5
-0.0024905676933988	7.5152392826124581	2.5431993187757480	W6
6.4898637667471686	1.8905833360312501	2.5084124025336503	W7
4.7817534554993264	4.6399160056080513	2.4574617039696980	W8
3.2548166758807993	7.4938492624394613	2.5084124025336503	W9
1.6735213026544833	1.0177417721363460	4.2015469598243280	Se1
0.0907425019971346	3.7591950720909608	4.2015469598243280	Se2
-1.6274084598061447	6.5770924701378695	4.2530028962029585	Se3
4.8361118047578078	1.0177417721368069	4.2015469598243280	Se4
1.6720377530992381	6.4980787886662439	4.2015469598243280	Se5
8.1370415673192067	0.9395846184583855	4.2008382003609759	Se6
6.4188906055158901	3.7591950720918232	4.2015469598243280	Se7
4.8375953545144963	6.4980787886667049	4.2015469598243280	Se8
1.6521982338852799	0.9342205542943751	0.8182197222986081	Se9
0.0077494712073110	3.7824893617726425	0.8182197222986081	Se10
-1.6274084598061447	6.5770924701378695	0.8526944004242399	Se11
4.8574348735238360	0.9342205542947821	0.8182197222986081	Se12
3.2548165537564797	3.7583385442981574	0.6618924970618421	Se13
1.6103677910775533	6.5583057168265082	0.8182197222986081	Se14
8.1370415673192067	0.9395846184583855	0.8191211600880879	Se15
6.5018836363066761	3.7824893617735889	0.8182197222986081	Se16
4.8992653165384663	6.5583057168269869	0.8182197222986081	Se17

2Se Vacancy

Lattice Vectors

9.6320258038606479	-0.0000000000011976	0.0000000000000000
-4.8160129017791622	8.3415790360103177	0.0000000000000000
0.0000000000000000	0.0000000000000000	20.0000000000000000

Cartesian Coordinate

0.0127663720094489	1.8610549745221567	2.4553386409415618	W1
-1.6053376338903851	4.7622184850303313	2.5948820408454760	W2
-3.0998172321530206	7.3507330801649839	2.5948820408454760	W3
3.2106752679889698	1.9495820316982617	2.4526163010423820	W4
1.6883875004852280	4.5862617888252641	2.4526163010423820	W5
-0.1108580355341733	7.3507330801644342	2.5948820408454760	W6
6.4085841639651111	1.8610549745213023	2.4553386409415618	W7
4.7329630355879990	4.5862617888247676	2.4526163010423820	W8
3.2106752680876367	7.3999956603047155	2.4553386409415618	W9
1.6285134011499873	0.9875906244343630	4.1982436561441725	Se1

0.0641975958266854	3.6970650784093220	4.1982436561441725	Se2
-1.6053377542594975	6.4878947826144584	4.4197120506095180	Se3
4.7928368939922512	0.9875906244339695	4.1982436561441725	Se4
1.6463593422974774	6.4374496089890174	4.1982436561441725	Se5
8.0266880495003416	0.9268420919401348	4.1518230720186020	Se6
6.3571526994137528	3.6970650784085399	4.1982436561441725	Se7
4.7749909530422805	6.4374496089886284	4.1982436561441725	Se8
1.6773864103883191	0.8327656389639867	0.8077546177648280	Se9
-0.0454482701091297	3.8168028387065305	0.8077546177648280	Se10
4.7439638847482737	0.8327656389635462	0.8077546177648280	Se11
3.2106751476203712	3.7073684372773266	0.6511796907028621	Se12
1.4878404671189225	6.4725368341622129	0.8077546177648280	Se13
8.0266880495003416	0.9268420919401348	0.7327855547278260	Se14
6.4667985653539750	3.8168028387057205	0.8077546177648280	Se15
4.9335098282220384	6.4725368341619030	0.8077546177648280	Se16

2Ses Vacancy

Lattice Vectors

9.6609213627883292	-0.0000000000006091	0.0000000000000000
-4.8304606812420356	8.3666033240975413	0.0000000000000000
0.0000000000000000	0.0000000000000000	20.0000000000000000

Cartesian Coordinate

0.0212482851298614	1.8715129624816058	2.5000000000000000	W1
-1.6101535603801898	4.6496196210343133	2.5000000000000000	W2
-3.2190023782022892	7.4362275152734423	2.5000000000000000	W3
3.2203071209679752	2.1065547160923255	2.5000000000000000	W4
1.8243298326729871	4.5244583056955543	2.5000000000000000	W5
-0.0013047424566839	7.4362275152732389	2.5000000000000000	W6
6.4193659567970958	1.8715129624811431	2.5000000000000000	W7
4.6162844093502065	4.5244583056950223	2.5000000000000000	W8
3.2203071210641774	7.4124454025200937	2.5000000000000000	W9
1.6756670123529158	1.0079019427386484	4.1931929938674282	Se1
0.1005485924564207	3.7360870740095602	4.1931929938674282	Se2
-1.6101536811078865	6.5073581177240296	4.2055446932459981	Se3
4.7649469880196147	1.0079019427383942	4.1931929938674282	Se4
1.6451885803383655	6.4114820123262399	4.1931929938674282	Se5
8.0507676815789893	0.9296225683252819	4.2071778423547821	Se6
6.3400654080153220	3.7360870740091077	4.1931929938674282	Se7
4.7954254202305329	6.4114820123259824	4.1931929938674282	Se8
1.6756670123529158	1.0079019427386484	0.8068071261325820	Se9
0.1005485924564207	3.7360870740095602	0.8068071261325820	Se10
-1.6101536811078865	6.5073581177240296	0.7944554267540120	Se11
4.7649469880196147	1.0079019427383942	0.8068071261325820	Se12
1.6451885803383655	6.4114820123262399	0.8068071261325820	Se13
8.0507676815789893	0.9296225683252819	0.7928222776452281	Se14
6.3400654080153220	3.7360870740091077	0.8068071261325820	Se15
4.7954254202305329	6.4114820123259824	0.8068071261325820	Se16

W Vacancy

Lattice Vectors

9.8949506060137598	0.0000000000039254	0.0000000000000000

-4.9474753028471294	8.5692785939538059	0.0000000000000000	
0.0000000000000000	0.0000000000000000	20.0000000000000000	

Cartesian Coordinate

-0.0760635115966075	1.8433042861417590	2.5000000000000000	W1
-1.7400003533023596	4.7253274366392883	2.5000000000000000	W2
-3.2983168685292421	7.6171366060350039	2.5000000000000000	W3
3.3743803803366106	1.8433042861431279	2.5000000000000000	W4
-0.0147784072800086	7.7134995013714542	2.5000000000000000	W5
6.5966337373779833	1.9042842100684370	2.5000000000000000	W6
5.0383172221497468	4.7253274366419777	2.5000000000000000	W7
3.3130952762388794	7.7134995013727741	2.5000000000000000	W8
1.6491583106670484	0.9808619004298483	4.1660789600146577	Se1
0.0011595628923584	3.8092377854945001	4.0886202000714880	Se2
-1.6242864310714609	6.6506345090425434	4.1660789600146577	Se3
4.9819748510901913	0.9720604369664305	4.2586114993198985	Se4
3.2971570585471923	3.8092377854956845	4.0886202000714880	Se5
1.6491583107729180	6.6636553475273477	4.0886202000714880	Se6
8.2112923762572674	0.9720604369677116	4.2586114993198985	Se7
6.5966336137258654	3.7687314506335712	4.2586114993198985	Se8
4.9226030526169531	6.6506345090451404	4.1660789600146577	Se9
1.6491583106670484	0.9808619004298483	0.8339211599853520	Se10
0.0011595628923584	3.8092377854945001	0.9113799199285221	Se11
-1.6242864310714609	6.6506345090425434	0.8339211599853520	Se12
4.9819748510901913	0.9720604369664305	0.7413886206801119	Se13
3.2971570585471923	3.8092377854956845	0.9113799199285221	Se14
1.6491583107729180	6.6636553475273477	0.9113799199285221	Se15
8.2112923762572674	0.9720604369677116	0.7413886206801119	Se16
6.5966336137258654	3.7687314506335712	0.7413886206801119	Se17
4.9226030526169531	6.6506345090451404	0.8339211599853520	Se18

WSe3 Vacancy

Lattice Vectors

9.7868425635624341	0.0000000000018304	0.0000000000000000	
-4.8934212816249856	8.4756542828400452	0.0000000000000000	
0.0000000000000000	0.0000000000000000	20.0000000000000000	

Cartesian Coordinate

-0.0690889489105019	1.8335728064940131	2.4383569283661402	W1
-1.7089047656633456	4.6738171162409818	2.4383569283661402	W2
-3.2622808543819284	7.5339149953028510	2.5560615151841541	W3
3.3313698034990367	1.8335728064947094	2.4383569283661402	W4
-0.0086753894042415	7.6187007803341542	2.4383569283661402	W5
6.5245617090763179	1.8834788067440613	2.4689699410313883	W6
4.9711856203563460	4.6738171162422315	2.4383569283661402	W7
3.2709562442059461	7.6187007803346480	2.4383569283661402	W8
1.6311403049462321	1.1520211343015765	4.1983538624505519	Se1
-1.4490311747107731	6.4870346332347300	4.1983538624505519	Se2
4.9178789028413368	0.9558600259405590	4.2046084715164120	Se3
8.1312442706060502	0.9558600259411599	4.2046084715164120	Se4
6.5245615867749889	3.7387160660521346	4.2046084715164120	Se5
4.7113117847996042	6.4870346332357007	4.1983538624505519	Se6
1.6311403049414255	0.8969501004814524	0.8168156173331560	Se7

0.1434805599529327	3.8497960459983869	1.1222565598167160	Se8
-1.6699291697673115	6.6145701501446608	0.8168156173331560	Se9
4.8927913115996393	0.9413756983840681	0.7062766161252200	Se10
3.1188000500387791	3.8497960459988221	1.1222565598167160	Se11
1.6311403050432118	6.4264983087749199	1.1222565598167160	Se12
8.1563318618471801	0.9413756983847387	0.7062766161252200	Se13
6.5245615867755227	3.7676847211651072	0.7062766161252200	Se14
4.9322097798609494	6.6145701501458953	0.8168156173331560	Se15

WSe6 Vacancy

Lattice Vectors

9.3528778482941828	-0.0000000000072522	0.0000000000000000
-4.6764389240055833	8.0998298150823445	0.0000000000000000
0.0000000000000000	0.0000000000000000	20.0000000000000000

Cartesian Coordinate

-0.2233890609370346	1.8991424588592776	2.5000000000000000	W1
-1.5846149289684446	4.2568548229283047	2.5000000000000000	W2
-3.1176259493055989	7.1998487983152639	2.5000000000000000	W3
3.3410150104350080	1.8991424588566288	2.5000000000000000	W4
0.1975871067716053	7.3437192980737178	2.5000000000000000	W5
6.2352518988942123	1.7999622549222185	2.5000000000000000	W6
4.7022408785483343	4.2568548229233141	2.5000000000000000	W7
2.9200388429163739	7.3437192980713188	2.5000000000000000	W8
1.5588128578255065	1.1828688725089296	4.1504904544824219	Se1
-1.3138250664474613	6.1584237092289840	4.1504904544824219	Se2
4.6794481202575344	0.9017184957350439	4.3441245811393960	Se3
7.7910554436775392	0.9017184957326886	4.3441245811393960	Se4
6.2352517820146645	3.5964494844049977	4.3441245811393960	Se5
4.4314507822719609	6.1584237092243557	4.1504904544824219	Se6
1.5588128578255065	1.1828688725089296	0.8495096655175880	Se7
-1.3138250664474613	6.1584237092289840	0.8495096655175880	Se8
4.6794481202575344	0.9017184957350439	0.6558755388606140	Se9
7.7910554436775392	0.9017184957326886	0.6558755388606140	Se10
6.2352517820146645	3.5964494844049977	0.6558755388606140	Se11
4.4314507822719609	6.1584237092243557	0.8495096655175880	Se12
